# Acute Cholecystitis in Aged Patients

**DOI:** 10.1155/1996/57138

**Published:** 1996

**Authors:** J. Prousalidis, E. Fahadidis, S. Apostolidis, C. Katsohis, H. Aletras

**Affiliations:** A.Prop. Surg. Clinic Department of Medicine Aristotles University of Thessaloniki AHEPA Hospital Thessaloniki Greece

## Abstract

The aim ofthis study is the analysis ofthe results in 62 patients over 70 years ofage with acute cholecystitis treated in our Department from 1970 to 1990. The clinical picture in 47 patients was mild and in 15 severe. In 14 cases (10 calculous, 4 acalculous) the acute cholecystitis subsided with antibiotics (Group A). In 48 more cases (45 calculous, 3 acalculous) following 1-3 days conservative treatment, operation was undertaken. Besides acute cholystitis there was gangrene of gallbladder in 10, choledocholithiasis in 7 and choloperitoneum without perforation in 7 cases. Cholecystostomy in 25, cholecystectomy in 15 and cholecystectomy with exploration ofthe bill duct in 8 cases was performed (Group B). There was one death in group A and 3 deaths .in group B. The hospital stay was 20 days. In conclusion the clinical findings in acute cholecystitis in the aged are usually mild. In the case of failure of medical treatment, after 2-3 days emergency surgery should be performed.


*HPB Surgery*, 1996, Vol. 9, pp.129–131
Reprints available directly from the publisher
Photocopying permitted by license only

© 1996 OPA (Overseas Publishers Association)
Amsterdam B.V. Published in The Netherlands
by Harwood Academic Publishers GmbH
Printed in Malaysia

### Acute Cholecystitis in Aged Patients

J. PROUSALIDIS, E. FAHADIDIS, S. APOSTOLIDIS, C. KATSOHIS
and H. ALETRAS

A.Prop. Surg. Clinic, Department of Medicine, Aristotles University of Thessaloniki,
AHEPA Hospital,Thessaloniki, Greece

*(Received 28 October 1993)*

The aim of this study is the analysis of the results in 62 patients over 70 years of age with acute cholecystitis treated in our Department from 1970 to 1990. The clinical picture in 47 patients was mild and in 15 severe. In 14 cases (10 calculous, 4 acalculous) the acute cholecystitis subsided with antibiotics (Group A). In 48 more cases (45 calculous, 3 acalculous) following 1–3 days conservative treatment, operation was undertaken. Besides acute cholystitis there was gangrene of gallbladder in 10, choledocholithiasis in 7 and choloperitoneum without perforation in 7 cases. Cholecystostomy in 25, cholecystectomy in 15 and cholecystectomy with exploration of the bill duct in 8 cases was performed (Group B). There was one death in group A and 3 deaths in group B. The hospital stay was 20 days. In conclusion the clinical findings in acute cholecystitis in the aged are usually mild. In the case of failure of medical treatment, after 2–3 days emergency surgery should be performed.

KEY WORDS: Acute cholecystitis

Gallstone disease in adults is a very common medical problem and surgical treatment is usually necessary (1). Acute cholecystitis is one of its complications reported frequently (2). This condition especially in the elderly is a serious and critical one with a significant morbidity (3). Due to the above reasons and to the increasing size of the elderly population, we are presenting our cases in order to draw some conclusions and make some proposals.

### MATERIALS AND METHODS, RESULTS

In the A' Propedeutic Surgical Clinic between 1970–1990, 1906 patients, 1567 under 70 years and 339 over 70 years, with gallbladder disease were admitted for treatment. Acute cholecystitis was noted in 234 cases of the first and 62 in the second aforementioned group which is the object of our study (Table I). Thirty three of the 62 aged patients were females and 29 were males. The age ranged from 70 to 95 with an average age of 77 years (Table II). Although information regarding the history was sometimes difficult to obtain, the mean duration of the biliary tract disease was 8 years. Fever of 37.4 to 38.5 C was present in all cases except one who had a temperature of 40°C. Dyspepsia and abdominal pain was reported in all cases. In 5 of

**Table I** Incidence of gallbladder disease and acute cholecystitis

| | | |
|---|---|---|
| Gallbladder disease | above 70 years | 339 |
| | under 70 years | 1567 |
| Total | | 1906 |
| Acute cholecystitis | above 70 years | 62 |
| | under 70 years | 234 |
| Total | | 296 |

**Table II** Sex and age distribution in 62 patients with acute cholecystitis

| | *Patients* |
|---|---|
| Females | 33 |
| Males | 29 |
| Range of age 70–95 years | |
| Average of age 77 years | |

*Correspondence to:* DR J. Prousalidis A'Prop. Surgical Clinic, AHEPA Hospital, Stilp. Kiriakidi 1, 54636 Thessaloniki, Greece them the symptoms were insignificant. Although the pain was sometimes difficult to define, it was described as a vague discomfort in the right side of the abdomen (40 cases), confined to the right upper quadrant (10 cases) and continuous and diffuse (12 cases). Jaundice was noted in 10 cases. Abdominal tenderness was noted as being mild (47 cases) or severe (15 cases). The tenderness was localized in the right abdomen in 50 cases and generalized in 12 cases. Leukocytosis was found in 40 patients with a range from 9800 to 22000 (average 12000). In the remainder of the cases, the white cell count was normal (Table III). Abnormal serum levels of bilirubin (10 cases), alkaline phosphatase (6 cases), transaminases (5 cases), and amylase (2 cases) were noted. Sonography in 17 cases and computed tomography in 4 cases supported the diagnosis.

**Table III** Presenting findings in 63 aged patients with acute cholecystitis

| | *Cases* |
|---|---|
| Fever | 62 |
| 37.4–38.5 C | 61 |
| 40 C | 1 |
| Dyspepsia | 62 |
| Abdominal pain | 62 |
| Discomfort | 40 |
| Colichy | 10 |
| Diffuse | 12 |
| Abdominal tendernes | 62 |
| Mild | 47 |
| Severe | 15 |
| Localized | 50 |
| Generalized | 12 |
| Jaundice | 10 |
| Leukocytosis | 40 |

Fifty five patients, were found to have calculous cholecystitis and seven acalculous cholecystitis. The clinical picture was comparatively more severe in the first than in the second group of cases. The diagnosis in the acalculous group was dependent only on the clinical picture without isolation of any precipitating factor. A history of systematic illness was reported in 21 cases (8 with diabetes, 2 with lung disease, 11 with heart insufficiency) (Table IV).

The acute cholecystitis in 14 cases (10 calculous, 4 acalculous) were managed nonoperatively with nasogastric suction, antibiotics, and intravenous fluids. Forty eight cases (45 calculous, 3 acalculous) required surgery. The timing of operation was 1–3 days after admission, except two cases with a delay of 4 and 6 days respectively. The preoperative diagnosis was correct in 45 cases. Cholecystostomy was done in 25 cases and cholecystectomy as a single procedure in 15 cases (Table V). Bile duct stones were noted in 7 cases, gangrene of the gallbladder with perforation and bile peritonitis in 10 cases and choleperitoneum without apparent perforation in 7 cases, (Table VI). In all these cases the inflammatory process was related to stones (calculous cholecystitis). In the conservatively treated group one death and in the surgically managed group three deaths occurred. The cause of death in the patient of the first group was stroke. The caüse of death in the three patients of the second group were septic shock, pneumonia and pulmonary embolism respectively (Table VII). In two of these three patients the surgery was delayed 4 and 6 days respectively. In the remainder of the operated patients the mean hospital stay was 18 days. The postoperative complications were of minor importance and did not increase the hospitalization.

**Table IV** Medical problems in 62 aged patients with acute cholecystitis

| | *Cases* |
|---|---|
| Diabetes | 8 |
| Lung disease | 2 |
| Heart insufficiency | *11* |
| Total | 21 |

**Table V** Management in 62 aged patients with acute cholecystitis

| | *Cases* |
|---|---|
| –Nonoperative | 14 |
| Calculous | 10 |
| Acalculous | 4 |
| –Operative | 48 |
| Cholecystostomy | 25 |
| Cholecystectomy | 15 |
| Cholecystectomy+common duct exploration | 8 |
| Calculous | 45 |
| Acalculous | 3 |

**Table VI** Complicated form of acute cholecystitis in 62 aged patients

| | *Cases* |
|---|---|
| Bile duct stoyes | 7 |
| Bile peritonitis | 17 |
| –with perforation | 10 |
| –without perforation | 7 |

**Table VII** Cause of death in 4 patients among 62 elderly cases with acute cholecystitis

| | *Patients* |
|---|---|
| Concervatively treated | |
| –stroke | 1 |
| Operatively treated | |
| –septic shock | 1 |
| –pneumonia | 1 |
| –pulmonary embolism | 1 |

### DISCUSSION

Acute inflammation of the gallbladder in the adult population has been well described by many writers (4). This paper deals with acute cholecystitis especially in the elderly because the disease in this age group is associated with a relatively poor prognosis (3, 4). The overall rate of acute cholecystitis is high (approximately 20% of cases of gallbladder disease) (2, 3). This incidence in patients 75 years of age or older, rises slightly (2) or to the rate of 31% or more (5). The rate for young to older patients is 3:1 (3). In our series, the percentage of acute cholecystitis under and above 70 years of age was 14.9% and 18.9% respectively. Although gallstones disease is consistantly more common in females, this proportion decreases with age (6). In our cases with acute cholecystitis, the females only slightly outnumbered the males.

The diagnosis of acute cholecystitis in this age group is often difficult and therapy is therefore delayed, increasing the morbidity and mortality (4, 7). In other series as well as in ours, the history is sometimes difficult to obtain. The peritoneal irritation may be absent or mild, and fever, white cell count and biochemical profile in a number of cases, are misleading (3, 4, 5).

According to the existing bibliography and our study, the cause of acute cholecystitis is more frequently cholelithiasis (calculous form) and rarely other causes (acalculous form) (8, 9). In our series of patients with the acalculous form of cholecystitis, no clear causative factors were found. Although the clinical picture in acalculous cholecystitis was less severe than its calculous form, both had similar presentation and were indistinguishable. Certainly the presence of bile duct stones, in calculous cholecystitis encountered frequently by others (5, 10) and in our study, gives a characteristic clinical picture.

The management of acute cholecystitis in the elderly is generally difficult. The major problem is the reluctance of the surgeon to operate on this population (3, 10). The reason for this attitude is the existence of other medical problems as reported by others (3, 5, 7) and noted in our group. This policy is dangerous because the inflammatory process in the gallbladder can sometimes lead to bile peritonitis from gangrene and perforation or to choloperitoneum without apparent perforation, especially in the elderly[4,11]. Although all but one of our conservatively treated patients did well, the medical management of acute cholecystitis in the elderly is reported to be unsuccessful (3). The delay in operation most probably played a role in the two deaths which occured in the surgically treated patients. Instead of cholecystectomy, high risk elderly patients can undergo cholecystostomy and keep complications at a minimum.

The mortality rate in emergency cholecystectomy, in the elderly, is between 9, 2% and 14% (3, 5, 12). In this study the relative rate in the operated group was 6%. The deaths occured in three patients with acute calculous cholecystitis and bile peritonitis.

In conclusion according to other authors and our limited experience, acute cholecystitis in aged patients should be treated for 1–3 days conservatively and in the case of failure, surgically[10,13]. Despite the existence of other medical problems in this age group, the policy needs to be energetic thereby preventing bile peritonitis or other complications.

### REFERENCES

1. Bouchier I.A.D. 1988. Gallstones: Formation and Epidemiology in Blumgart L.H. Surgery of the Liver and Biliary Tract Vol I pp 503–516 Churchill Livingstone.
2. Hermann R.E. Grundfest-Broniatovski S. 1988. Chronic Cholecystitis in Blumgart L.H. Surgery of the Liver and Biliary Tract Vol I pp 541–549 Churchill Liningstone.
3. Glenn F., 1981. Surgical management of acute cholecystitis in patients 65 years of age and older. *Ann. Surg.* **193** : 56–59.
4. Cushieri A., 1988. Acute cholecystitis in Blumgart L.H. Surgery of the Liver and Biliary Tract Vol. I pp 531–539.
5. Huber D.F., Martin E.W.Jr., Cooperman M., 1983. Cholecystectomy in Elderly Patients *Am. J. Surgery* **146**: 719–722.
6. Bateson M.C., Bouchier I.A.D., 1975. Prevalence of Gallstones in Dundee: A Necrophy Study. *Br. Med. Journal* **4**: 427–429.
7. Glenn F., 1976. Acute Cholecystitis S.G.O. **143**:56–60.
8. Keddie N.C., Gough A.L., Galland R.B., 1976. Acalculous Gallbladder Disease: A Prospective study *Br. J. Surgery* **63** :797–798.
9. Robertson R.D., 1970. Noncalculous Acute Cholecystitis Following Surgery. Trauma and Illness *Am. Surg.* **36**: 610–614.
10. Sullivan D.M., Fuffin Hood T., Griffey W.O., 1982. Biliary Tract Surgery in the Elderly. *Amer. J. Surgery* **143**: 218–22.
11. Kent S.J.A., and Mentzies-Gow N., 1974. Biliary Peritonotis without Perforation of the Gallbladder in Acute Cholecystitis *Br. J. Surg.* **61**: 690–692.
12. Morrow D.J., Thompson J., Wilson S.E., 1978. Acute Cholecystitis in the Elderly *Arch. Surg.* **113**: 1149–1152.
13. Ljunodali E.G. 1990. Acute Cholecystitis in the elderly. *Am. J. Surg.* **159**: 414–416.

